# Biomechanical, Topological and Chemical Features That Influence the Implant Success of an Urogynecological Mesh: A Review

**DOI:** 10.1155/2016/1267521

**Published:** 2016-04-28

**Authors:** Carmelo De Maria, Vito Santoro, Giovanni Vozzi

**Affiliations:** ^1^Research Center “E. Piaggio”, University of Pisa, Largo Lucio Lazzarino 2, 56122 Pisa, Italy; ^2^Dipartimento di Ingegneria dell'Informazione, University of Pisa, Largo Lucio Lazzarino 2, 56122 Pisa, Italy

## Abstract

Synthetic meshes are normally used to treat several diseases in the field of urogynecological surgery. Not-optimal selection of mesh and/or its not-correct implant may increase patient's pain and discomfort. The knowledge of mechanical behaviour and topological and chemical properties of a mesh plays a fundamental role to minimize patient's suffering and maximize the implant success. We analysed several papers reporting the meshes application for urogynecological pathologies, to extrapolate the principal parameters that normally are used to characterise the biomechanical, topological, and chemical properties, and to verify their influence on implant success. In this way we want demonstrate that, knowing these features, it is possible to foresee the success of a mesh implant. This review shows that the application of a mesh strictly depends on elastic modulus, failure load, porosity and pore size, filament diameter, polymer weight, and crystallinity. To increase the success of the implant and to help choice of optimal mesh for a clinical need, two indexes have been proposed for comparing, in an easier way, the mechanical performance of different commercially available meshes.

## 1. Introduction

Nowadays, polypropylene (PP) synthetic meshes are commonly used in many urogynecological surgical procedures, such as sacrocolpopexy, anterior-posterior pelvic organ prolapse (POP), and suburethral sling for urinary incontinence treatment.

POP is one of major problems that occur in more than 50% of women after childbirth and it may be treated [1] with several surgical procedures. Nevertheless, further postsurgical prolapse or recurrences are observed due to the increase of average life expectancy. The longevity determines a progressive deterioration of type I collagen, which is gradually replaced by less resistant type III collagen. Several studies reveal that the pelvic organ prolapse is either caused by excessive pressure within endopelvic fascia or fascial disruptions that require appropriate* in situ* reinforcements [2]. Normally the fascia takes about 3 months to recover 70% of its natural resistance [3]. The main goals of prosthetic surgery are the complete reconstruction of pelvic floor, the restoration of normal anatomy and function, the absence of tension on the vaginal wall (cause of pain) and of complications (e.g., infection or allergic reactions), and a high degree of satisfaction by patients.

The implant of biocompatible synthetic meshes significantly improves the restoration of anatomy of anterior vaginal wall. However, a high rate of complications, including 10% erosion, is a matter of concern. In addition, significant changes into pelvic wall structure can be evaluated by palpating the surface of the vaginal epithelium that appears rigid. Generally, the implant sites have low elongation capacity and flexibility which can lead to pain, discomfort, and dyspareunia in human body: these indications mean that prosthesis is not completely compatible in terms of mechanical properties of natural tissue [4].

Benson et al. [5] show that the surgical technique concerning the use of meshes has better outcomes within apical vaginal prolapse compared to vaginal surgery with a sacrospinous fixation. A large amount of papers support the use of mesh in surgery; in particular, the guidelines for vaginal prolapse surgery show that meshes are better performing with respect to other traditional techniques [6]. In comparison to old techniques the innovative surgical procedure for transvaginal prolapse (Apogee, Perigee, and Prolift) has shown good results in short term, despite having significant complications such as buttock pain, vaginal erosion, erosion of the bladder, and infection and therefore should be used carefully. Some of these consequences could be related also to the surgical procedure, such as the mesh preloading; in other cases, the intraoperative retraction of the mesh could be misinterpreted as shrinkage [7].

Nevertheless, the use of polymeric meshes presents best results in terms of permanence and success of reconstructive procedures that currently have a failure rate greater than 30% using the traditional methods [8]. This high failure rate is due to the fragility of endogenous tissue in female patients with prolapse as reported in several reports [9, 10]. A good biomechanical integration of mesh with the pelvic tissue is the fundamental property that any prosthesis used in urogynecology and andrology should have. As well-known from literature, a rigid material can develop an excessive stress at the interface inducing prosthesis erosion and tissue exposure [11]. Parameters such as mesh size, regrowth inside its fabrics, its mechanical, chemical, and physical properties, and anchoring technique play an important role on the success of a prosthetic implant [12]. The mechanical properties should be comparable to that of natural tissue, and meshes should be stable for a long time showing resistance to shrinkage. Pore size highly influences the success of implant; a pore size greater than 75 *μ*m encourages regrowth of blood vessels, fibroblast colonization, and collagen production [13].

Therefore, several complications are closely related to mesh features such as topology, porosity, stiffness, and filament composition.

**Figure 1:**
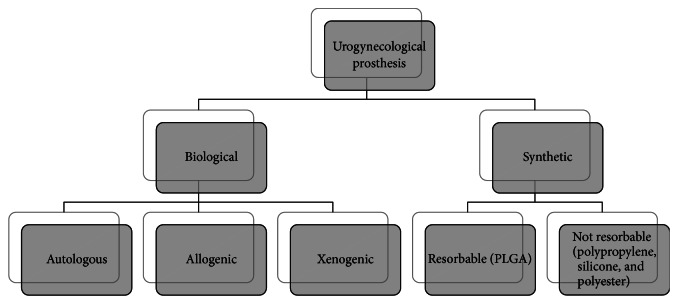
Classification of urogynecological prosthesis.

A wide range of meshes is currently available to clinicians for urogynecological and andrological surgery. Generally, it is possible to classify the products on the basis of the biomaterial used (Figure 1). Biological prostheses have the theoretical advantage of reducing the tissue erosion rate but in the same time they present several disadvantages such as mechanical inconsistency and potential transmission of infections. Furthermore, autologous implants require presurgical procedure into patients increasing their suffering. This last problem is overcome by allografts prosthesis where tissues are harvested from cadavers and biomechanically tested before their use, but often it is difficult to find donors or the explanted tissue has no right features for the implantation. Xenografts are easily available even if different studies show the presence of an excessive inflammatory reaction which can lead to rejection process. Acellular xenogeneic collagen matrix transplants, used to repair advanced prolapse, have not produced the desired results considering the high rate of failure due to postsurgical procedures complications [14]. Then, to overcome the limits and problems due to prosthesis produced by natural tissues, several meshes made of absorbable polymers have been tested but their inefficiency for urogynecological implants has been demonstrated [15]; cells colonize these structure and start to restore the damaged tissue with a reduced inflammatory response, but their degradation time is less than of that of tissue restoration, so their support is not sufficient to ultimate the recovery of natural tissue. For these reasons, the nonresorbable synthetic meshes are considered ideal for reconstructive surgery of the pelvic tissues.

Unfortunately, the newly formed tissue is often atrophic and without vascular network, causing poor tissues regeneration with risks of inadequate healing and mesh exposure.

Several studies present in the literature show that* in vivo* dimensional changes of mesh are the main cause of stiffness increase and low restore of vaginal tissues standard properties [4]. None of commercially available materials satisfies all the requirements [16, 17]. Nylon, Marlex, and Gore-Tex meshes have higher erosion rates, higher stiffness, and also substantial differences in pore size, in manufacturing process, in surface properties, and in mesh topology [7] compared to PP meshes currently marketed. For these reasons PP mesh is considered the gold standard for urogynecological treatment.

The PP meshes erosion rate in surgery for stress incontinence is around 1–3% [18]. Synthetic meshes made of other polymers have a low elasticity, normally with pore size less than 10 *μ*m and multifilament weft. These features may predispose the patient to erosion and pain (17–20%) [19, 20]. From this point of view, PP meshes appear to have better characteristics of resistance and elasticity, but their values do not match those of surrounding tissues and* in situ* integration could be affected by this difference [21, 22].

The problems associated with the surgical use of mesh for pelvic organ prolapse vary drastically from small erosions to perforations in bladder and intestines. The list of complications includes acute and chronic infections, tissue contraction due to mesh shrinkage, erosion of tissue adjacent to the mesh, pain, and dyspareunia, and limitation of sexual activity [23–26]. In Table 1, the literature data are reported.

Summarising, the type of material that composes the meshes and their biomechanical and topological features, plays an important role in the tissue regeneration process and consequently in the implant success, as highlighted by the present review. In addition, we propose the use of two indexes for classifying commercial available meshes, for indicating possible design direction, and for helping the surgeons in their choice on the basis of their clinical needs to maximize the implant success.

## 2. Biomechanical Properties of Commercial Urogynecological Mesh

The optimal biomechanical properties that prosthesis for urogynecological surgery should present [27, 28] are not well defined yet. The mechanical behaviour of these meshes depends on the polymer in which they are made as well as on the type of fiber used, their weft, and pore size [4]. Stiffness, relative elongation, and failure load are the principal parameters that characterise the biomechanical behaviour of a mesh. In particular, the mesh stiffness is the factor closely linked to tissue erosion, mesh exposure, and pain. It depends on many factors such as mass per unit area, weft structure, working technique used to fabricate the mesh, and pore size [12].

**Figure 2:**
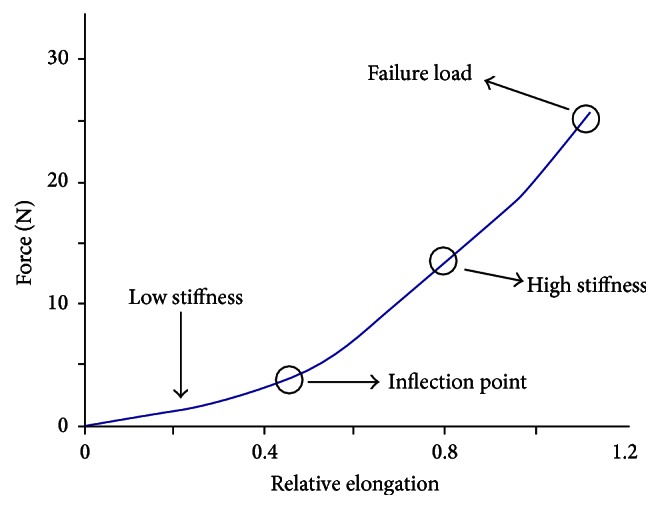
Typical force: relative elongation graph of a synthetic mesh.

In the literature different types of slings for incontinence, which have similar weights but fully different biomechanical behaviour and thus different functionality, are reported [16, 29]. Biomechanical behaviour of meshes is commonly evaluated using uniaxial test, assessed on sterile samples cut in strips. The strip length is bigger than width in order to minimize the effects of nonlinearity. This is due to the clamps of mechanical testing system [4–12]. Before mechanical assessment, each sample is dipped in a physiological solution bath at 37°C for 10 minutes and then the mechanical test is performed in wet conditions. On the sample a preload of 0.1 N is applied. The displacement rate is set up to 50 mm/min until the probe is broken. The acquired data allows to determine the stress-strain curve in which distinguishing two different regions is possible: the initial one with a low stiffness due to stretching of mesh weft and a second region with a high stiffness due to polymer mesh (Figure 2) [12]. The low and high stiffness regions are defined as the minimum slope over a 15% and 30% relative elongation, respectively. The inflection point is defined as the intercept of the two tangents of stress-strain curve in the two previous regions. Within high stiffness region the registered loads overcome the forces that normally act in physiological conditions (*in situ*) [4], which are more similar to those present in low stiffness area.

In Table 2 the mechanical properties of several commercially available meshes are reported: Caldera Ascend mesh has the highest value between low and high stiffness region.

These data could be difficult to be understood by surgeons: it is important to determine an index that can be easily read. Furthermore, biological tissues present an anisotropic behaviour, which should be taken into account in the prosthesis design. In the field of hernia repair, the anisotropy index *λ* was proposed [30, 31] to describe the different mechanical behaviour along the two different tensile directions in the mesh plane. It is defined as

(1) $$\begin{array}{c} \lambda =\left|\;\log\frac{E_{L}}{E_{T}}\;\right|, \end{array}$$

where *E*_*L*_ and *E*_*T*_ are the elastic modulus in longitudinal and transverse direction, respectively. Once the mechanical properties have been quantified in two directions, this index allows to compare the mesh behaviour with the target tissue.

The forces that meshes are able to support, reported in Table 2, depend directly on their structural elasticity and are essential for the stability of the implant. Also this parameter is directly related to mesh and tissue erosion, mesh exposure, and pain [12].

For this reason it is important also to determine its failure load and relative elongation at inflection point. Dietz et al. showed [16] that mechanical properties of urogynecological implants should be related to the range of human physiological strength.

For the abdominal wall meshes, the security index *K* has been defined [30] to evaluate if a mesh is able to support the forces that are generated* in situ*; it is defined as

(2) $$\begin{array}{c} K=\frac{\sigma_{m}}{\sigma_{tissue}}, \end{array}$$

where *σ*_*m*_ is the maximum stress which can be sustained by the surgical meshes and *σ*_tissue_ is the typical stress of the specific tissue. In the case of urogynecological meshes, as precautionary, the same stress used for abdominal wall could be considered [31].

Table 3 shows the security index of different commercial meshes. The bigger the index the less the probability that the mesh is broken by forces acting normally on natural tissue.

Once implanted the mesh must hold its shape and position and must resist different stresses. These can be either raised during surgical procedure or during the patient life. Meshes should present a high stability in an environment with a pH close to 7.0, PO_2_ less than 40 mmHg, and temperature between 28 and 37°C [32]. A variation of tensile strength of mesh and an increase of its elongation may cause recurrences.

The long term stability of PP has been tested in several works: experimental results indicate a degradation of PP, with consequent reduction of mechanical resistance [30, 33, 34]. The chemical structure of PP had multiple functional groups that were potential sites for chemical reactions. The carbon-carbon backbone was not well shielded since the hydrogen and methyl groups did not pack tightly together.

## 3. Topological Parameters of Commercial Urogynecological Mesh

Filament diameter, pore size, and porosity play a fundamental role in the development and treatment of complications related to the use of synthetic meshes.

The filament, usually made in PP known also with its commercial name of Prolene® or Marlex® [35], has a diameter which varies within the range of 0.08 mm to 0.66 mm. This geometrical feature contributes to the formation of fibrotic tissue and tissue integration of mesh, as well as to the success implant, especially in the case of vaginal prolapse [36]. The ideal products for urogynecological surgery are made of monofilament fibers and with large pore size (>75 *μ*m) which allow low rates of infection and erosion. This pore size enables the passage of macrophages, fibroblast colonization, a rapid regrowth of blood vessels (angiogenesis), and collagen production. The inflammatory response is stopped quickly by allowing the mesh to be incorporated by fibrous tissue, preventing the granuloma formation. Granuloma develops around the single fibers of mesh as a result of foreign body reaction and can lead to infection, erosion, and inflammation of the tissue in contact with it [4, 36]. If the mesh has pores smaller than 800 nm, the possibility of granuloma development is higher and it encapsulates synthetic structure, creating a planar scar and reducing inherent flexibility [29–36].

The porosity and the mean filament diameter of different meshes (evaluated processing optical and SEM microscopy images) are reported in Table 4 [4–12]. There parameters vary over a wide range.

## 4. Chemical Parameters of Commercial Urogynecological Mesh

In general, considering the weight of commercial products, it is possible to distinguish in two types of mesh: heavy and light meshes [37]. This weight depends on the used polymer and on the weft [36, 38]. The heavy meshes are usually made with a thick filament, presenting small pore size and high tensile strength. Usually the weight of these meshes is about 100 mg/mm^2^. The light mesh is usually made of thin filament and it presents large pores. These last meshes have an average weight of 33 mg/mm^2^, are elastic, and generate a lower foreign body reaction.

As reported in the literature, there is a strong positive correlation between the weight of the mesh and its tensile strength: lighter meshes support lower loads at the failure point [4]. However, tensile strength and elongation have magnitudes higher than those observed* in vivo*, for this reason the principle of* less foreign material in the body* is followed.

Finally, the polymer crystallinity increases also the strength as well as the stiffness of relative mesh.  Table 5 reports both weight and polymer crystallinity degree of commercial available meshes discussed in this review.

## 5. Conclusions

In this review the principal parameters that influence the success of a mesh implant for the cure of pathologies in urogynecological field have been analysed. We observed that the meshes normally used in this surgical area are made of synthetic polymers, principally PP, and that the tissue response and the damage repair can be related to mechanical properties (as elastic modulus, elongation, and failure load), filament diameter, porosity, polymer molecular weight, and crystallinity.

It is often difficult for a clinician choosing the best mesh for specific clinical needs; for this reason we have proposed two indexes (anisotropy index and security index) that allow to easily classify the mechanical performance of commercially available meshes and furnish a novel methodological approach to analyse their performance.

## Figures and Tables

**Table 1:** Percentage of principal complications reported in the literature.

Complications	Range based on clinical data (%)	Range based on random trials (%)
Erosion	1–18.8	5–19
Pelvic, groin, and buttocks pain	2.9–18.3	0–10
Dyspareunia	2.2–15	8–27.8
New surgical intervention	1.3–7.6	3.2–22

**Table 2:** Mechanical features of principal commercially available urogynecological meshes.

Mesh	Low stiffness (N/mm)	High stiffness (N/mm)	Relative elongation at inflection point (%)	Load at mesh failure (N)
AMS IntePro Lite*™*	0.071 ± 0.01	0.934 ± 0.04	33.9 ± 1.0	27.2 ± 1.9
Boston Scientific Polyform*™*	0.13 ± 0.01	1.42 ± 0.11	39.9 ± 1.5	53.8 ± 4.8
Caldera Ascend*™*	0.724 ± 0.2	1.66 ± 0.26	13.4 ± 2.1	41.1 ± 5.3
Coloplast NovaSilk*™*	0.072 ± 0.05	0.508 ± 0.09	44.6 ± 7.5	19.6 ± 4.5
Gynecare Gynemesh PS*™*	0.286 ± 0.02	1.37 ± 0.09	25 ± 0.89	46.3 ± 2.6
Mpathy Smartmesh*™*	0.178 ± 0.03	0.592 ± 0.04	29.2 ± 1.0	22.7 ± 1.9
Dipromed DAL3P	0.32 ± 0.05	1.18 ± 0.07	30 ± 2.5	60 ± 3.1
Dipromed EV3P	0.529 ± 0.023	0.7535 ± 0.3	80 ± 5.0	76.2 ± 5.2
Dipromed 120 ML	0.36 ± 0.03	0.588 ± 0.09	80 ± 5.0	89.6 ± 4.3

**Table 3:** Security index of principal commercially available urogynecological meshes (rounded by defect to eliminate the effect of uncertainty).

Mesh	Security index *K*
AMS IntePro Lite	2.3
Boston Scientific Polyform	4.6
Caldera Ascend	3.5
Coloplast NovaSilk	1.4
Gynecare Gynemesh PS*™*	4.0
Mpathy Smartmesh	1.9
Dipromed DAL3P	5.2
Dipromed EV3P	6.6
Dipromed 120 ML	7.7

**Table 4:** Topological features of principal commercially available urogynecological meshes.

Mesh	Mean diameter of filament (*μ*m)	Porosity (%)
AMS IntePro Lite	0.248	66.9 ± 0.96
Boston Scientific Polyform	0.66	56.09 ± 3.2
Caldera Ascend	0.248	51.3 ± 4.4
Coloplast NovaSilk	0.09	61.3 ± 3.8
Gynecare Gynemesh PS	0.094	62.1 ± 3.2
Mpathy Smartmesh	0.08	71.9 ± 1.4
Dipromed DAL3P	0.12	80.4 ± 2.2
Dipromed EV3P	0.12	88.0 ± 1.3
Dipromed 120 ML	0.12	90.0 ± 1.5

**Table 5:** Chemical features of principal commercially available urogynecological meshes.

Mesh	Weight (mg/mm^2^)	Crystallinity (%)
Aris	0.065	44.2
Autosuture	0.083	54.4
Avaulta	0.058	47.0
TVTO	0.093	49.1
Uretex	0.078	51.2
Dipromed DAL3P	0.063	47.4
Dipromed EV3P	0.050	47.4
Dipromed 120 ML	0.039	47.4
